# How Reliably Can We Measure a Child’s True IQ? Socio-Economic Status Can Explain Most of the Inter-Ethnic Differences in General Non-verbal Abilities

**DOI:** 10.3389/fpsyg.2020.02000

**Published:** 2020-08-07

**Authors:** Dacian Dolean, Alexandra Cãlugãr

**Affiliations:** ^1^Faculty of Psychology and Educational Sciences, Babes-Bolyai University, Cluj-Napoca, Romania; ^2^Cluj-Napoca Technical College, Cluj-Napoca, Romania

**Keywords:** Roma children, intelligence, socio-economic status, Raven Progressive Matrices, segregation

## Abstract

Roma children have been discriminated against for many years as they are denied access to high-quality education based on their scores on general non-verbal IQ tests. Rushton et al. (2007) showed that Roma perform more poorly than non-Roma on one such test (i.e., Raven Progressive Matrices), but suggest that this underperformance could be explained by Roma’s low socio-economic status. In this paper, we tested the non-verbal abilities of Roma children and expanded on the research of Rushton et al. (2007) by investigating empirically the potential mediating effects of socio-economic status on children’s performance on Raven Progressive Matrices. Results showed that the performance of Roma children was, on average, significantly lower than the performance of their non-Roma peers; however, the effect of ethnicity was partially mediated by the parents’ education and living conditions (while the parents’ income had no significant effect). As hypothesized by Rushton et al. (2007) some socio-economic factors can explain important variability in the performance of Roma children on general non-verbal tests, and their poor performance on such tests may lead to an underestimation of the true population mean.

## Introduction

Several studies have investigated the differences between different ethnic groups when it comes to their performance on general non-verbal abilities tasks (e.g., Herrnstein and Murray, 1994; Lynn, 2006; Lynn and Mikk, 2007). One such study, published in the journal *Intelligence* more than 10 years ago (Rushton et al., 2007) measured the non-verbal abilities of a large sample of Roma minority from Serbia and indicated that, on average, their IQ was significantly lower than that of their non-Roma European counterparts and similar to that of sub-Saharan groups. The study by Rushton et al. (2007) may fuel a bias in social comparison that could subject Roma people to discrimination (Schuch, 2016) based solely on the assessment of their cognitive abilities with an IQ test that is assumed to be culturally neutral. Not only does the study by Rushton et al. (2007) have the potential to support exclusionary practices against Roma (particularly children) that are reported in many European countries, but the conclusions of the study are questionable since the research did not account for many of the confounding variables that could explain the poor performance of Roma on the non-verbal test, including the socio-economic status of participants in the study. Since then, no additional study has been conducted to further investigate this issue, and it is not clear to what extent the performance of Roma on such assessments is a reliable indicator of their cognitive abilities, nor to what extent their performance is affected by socio-economic circumstances. In this short report, we extend Rushton et al.’s study by measuring the performance of an identically sized sample of Roma on the same general non-verbal abilities test (i.e., Raven Progressive Matrices) and comparing it with the performance of non-Roma from the same communities, while accounting for the socio-economic status of the participants.

### The Implications of the Assessment of General Non-verbal Abilities of Roma Children

Roma is an ethnic minority that has a long history of struggling with poverty, marginalization, discrimination, and social injustice (Schuch, 2016; Sutherland, 2017). One of the main discriminatory practices against Roma children has long been their segregation in schools that offer low quality educational programs, some of which are designed for students with intellectual disabilities (Save the Children, 2001; O’Nions, 2010; Brüggemann, 2012; White, 2012; FXB Center for Health and Human Rights at Harvard University, 2015; Cashman, 2016; Amnesty International and European Roma Rights Centre, 2017; Messing, 2017). The aforementioned studies show that the overrepresentation of Roma students in segregated schools is often explained by the fact that general IQ tests (such as Raven Progressive Matrices) are used to identify students with intellectual disabilities, and Roma children typically perform more poorly on such tests than do their non-Roma peers. However, the practice of using general intelligence tests to assess the eligibility of Roma children to attend high-quality schools is problematic for two reasons.

First, basing school placement solely or primarily on such intelligence tests may lead to social comparisons (Festinger, 1954) that have a negative effect on Roma children, as a group. In addition, the practice of excluding children from mainstream schools can detrimentally impact their identity formation (Erikson, 1968). In the case of Roma children, an early unsuccessful performance on such tests, coupled with a resulting placement in less competitive schools, can shape their identity through upward social comparisons with the majority of their non-Roma peers (e.g., “*We, Roma students, are not smart enough to attend the good schools that other students attend”*). Social comparison research on minority groups has indicated that group membership strongly influences a perceived similarity with other members of the group (i.e., assimilation on the basis of identity) (Brewer and Weber, 1994; Mussweiler and Bodenhausen, 2002). Research has also shown that comparisons with members from other groups can increase a perceived dissimilarity with individuals from the reference group (i.e., contrasting effects in self-evaluations) and can lead to self-stereotyping (Mussweiler and Bodenhausen, 2002; Mussweiler, 2003).

The results of such tests do not only affect the Roma children’s self-perception. Rather, these results may also encourage educational stakeholders to make downward social comparisons of Roma children as a group, thereby justifying their educational segregation by claiming that Roma children would be more likely to fail if they attended mainstream schools with a regular curriculum (e.g., *“These tests prove that many Roma children are not smart enough, and it is for their own good that they are placed in less competitive schools”*). In other words, instead of increasing efforts toward an inclusive education, some educational stakeholders could use the results of intelligence tests to justify their exclusion of Roma children from the mainstream schools.

Second, the usage of standardized intelligence tests as a selection criterion for the placement of Roma children in schools has already been heavily criticized because the tests are not as culture-free as they claim to be. Advocates for Roma children voice concerns that such tests can put Roma children at a disadvantage, as their performance can rely on contextual factors with which Roma children are not familiar. Therefore, using the results of these tests to place Roma students in schools with an abbreviated curriculum and low academic expectations is a discriminatory practice. Indeed, empirical evidence shows that intelligence tests are culturally loaded (Kan et al., 2013), and even the results of less culturally sensitive non-verbal tests such as Raven Progressive Matrices (RPM) are still biased by the background of their participants (Owen, 1992). The studies indicate that different contextual factors can lead to different patterns in children’s performances on general IQ tests. However, to date, there are no empirical studies to support the claim that Roma children might perform poorly on general non-verbal IQ tests due to the influence of certain contextual factors on their development.

One set of contextual factors known to correlate with IQ is the socio-economic status (SES) of the test taker. This construct is usually measured by several indicators, including education, income, and living conditions (Grusky, 2001). Multiple studies indicate that a significant amount of variance in the IQ of children is explained by the SES of their families, and the environmental influence on IQ is particularly strong among children raised in families with low SES (Heckman, 2006; Hanscombe et al., 2012; Von Stumm and Plomin, 2015). Thus, it is plausible to believe that the IQ scores of many Roma children can be at least partially explained by their typically low SES, although no study has empirically investigated this hypothesis.

### The Non-verbal Abilities of Roma

Several studies have measured the performance of Roma on IQ tests, with the most well-known research published by Rushton et al. (2007). In this study, the general intelligence of 323 Roma adults from 3 Serbian communities was measured using Raven’s Progressive Matrices. The authors justified their use of this test by citing its popularity and high psychometric properties among a large variety of cultural groups (Raven et al., 1991). The test measures general intelligence (the *g* factor) as described by Spearman (1927), with a focus on non-verbal skills and analogical thinking (Raven et al., 1998). The authors used both Colored Progressive Matrices (which are typically administered to children and/or adults with low intellectual ability) and Standard Progressive Matrices (which are typically administered to adults). The study was unique because it included a large sample size of Roma participants tested individually. The results indicated that Roma performed very poorly as compared with other European groups and performed similarly to Sub-Saharan groups. Although their findings, which indicated a poor performance of Roma on general IQ tests, are consistent with previously and subsequently published studies (Raven et al., 1998; Save the Children, 2001; Bakalar, 2004; Dolean and Tincas, 2018), the authors acknowledged several limitations of their study.

One limitation is the possibility that the scores of Roma might reflect the educational background of participants and “may seriously underestimate the true Roma population mean” (Rushton et al., 2007, p. 10). Their concern seems to be justified. A recently published meta-analysis from 42 data sets including 600,000 participants indicates that a longer education increases intelligence by 1 to 5 IQ points for each additional year of schooling (Ritchie and Tucker-Drob, 2018). The authors concluded that “education appears to be the most consistent, robust, and durable method yet to be identified for raising intelligence” (Ritchie and Tucker-Drob, 2018, p. 1). These results suggest that the Roma’s performance on IQ tests might be lower than that of their peers due to their low level of education. Consequently, accounting for the number of years of schooling of the parents of Roma children seems crucial to interpreting the results of their IQ tests, especially as recent findings have shown that SES explains an important variance in Roma children’s IQ (Lervåg et al., 2019) and parenting programs that aim to educate Roma parents about helping their children with schoolwork seem a feasible strategy for the alleviation of this disadvantage.

Another limitation of the study conducted by Rushton et al. (2007) is the possibility that Roma might perform poorly on IQ tests as a result of their poverty. Indeed, poverty has been found to impede IQ performance measured with RPM (Mani et al., 2013) because it is believed that the concerns related to poverty have adverse effects on mental resources. Furthermore, poverty can lead to a “sub-optimal level of nutrition that has an adverse effect on general intelligence” (Rushton et al., 2007, p. 10). Although the authors have not supported their assumptions with empirical evidence, subsequently published studies indicate that IQ varies based on the quality of the participants’ food intake (Von Stumm, 2012; Nyaradi et al., 2013; Robinson et al., 2018), and children raised in poverty have a low-quality diet (e.g., Leung et al., 2014). It is a plausible assumption that Roma’s substandard economic background may impede their access to nutritious food. Therefore, when we measure the IQ of Roma and compare it with the IQ of their non-Roma peers, it is essential to account for income as a potential confounding variable that can lead to unequal access to proper nutrition among participants, in addition to other poverty-related concerns that may explain lower scores on IQ tests. This is important because, if improper nutrition is a contributing factor to the poor performance of Roma children on such tests, developing school programs that provide Roma children with nutritious meals could potentially improve their test performance.

Finally, Rushton et al. (2007) stressed that Roma tend to live in inadequate housing conditions which might impede their cognitive development. The authors mentioned that “Roma children grow up in disadvantaged conditions, often live in overcrowded homes and are not as exposed to the intellectual stimulation and test taking attitudes typically associated with high test scores” (Rushton et al., 2007, p. 10). Indeed, empirical evidence suggests that inadequate physical environments (such as overcrowded homes) can have a detrimental effect on children’s cognitive development due to improper cognitive stimulation (Solari and Mare, 2012; Ferguson et al., 2013). Furthermore, overcrowded housing can adversely impact the home support network and the homework environment through phonological interference (Vasilev et al., 2018), which can, in turn, adversely impact the education of Roma children. Thus, in order to understand the underlying mechanisms that explain the performance of Roma on IQ tests, we also need to account for housing conditions as a potential confounding variable. This issue is important because, if quality of housing is a strong predictor of the children’s performance on non-verbal abilities tests, then social programs focused on improving the housing conditions of Roma children could have meaningful positive outcomes on their performance.

### The Present Study

In this study we aim to expand Rushton et al.’s findings by measuring the general intelligence (*g*) of an identically sized sample of Roma using the same test (RPM). However, unlike Rushton et al. (2007) whose study was focused on adults, our study will focus on children. We believe that studying children is particularly important given the context of school segregation justified by low scores on general IQ tests. While Rushton et al. (2007) did not use a control sample of non-Roma participants from the same communities (and instead compared the scores with the nationally normed scores of adults from Serbia), we compare the IQ of Roma children with the performance of their non-Roma peers residing in the same communities. We believe such comparison is important to minimize the potential effects of the socio-economic circumstances that may lead Roma children to perform poorly on IQ tests and to distinguish between such environmental factors and the actual cognitive abilities of Roma (as a distinct ethnic group). Furthermore, we address the acknowledged limitations of the Rushton et al. (2007) study by accounting for the following potential socio-economic variables that could explain a reported low performance on IQ tests: the parents’ education (both the mother’s and the father’s), wealth (income), and living conditions (the number of people per room living in one household). We hypothesize that the reported low performance of Roma children on RPM can be at least partially explained by these socio-economic factors.

We ask the following questions:

1. Is the IQ of Roma significantly lower than the IQ of non-Roma?
2. Can the potential differences in IQ be partially explained by differences in socio-economic status?

## Materials and Methods

### Data Collection Context

This study was part of a longitudinal research focused on the factors that contributed to the development of academic skills in Roma children from Romania. The research followed the ethical guidelines of the higher education institution of the first author. The data used in this study was collected in October 2014. The participating children were individually tested with multiple cognitive and academic measures, including the non-verbal abilities (see below) by trained research assistants with backgrounds in psychology and/or educational sciences. The children were tested in quiet rooms, in their schools. The socio-economic data was collected from parents by the classroom teachers during parent-teacher conferences. The teachers assisted the few parents whose literacy skills were too limited to independently complete the demographic data. Information regarding the children’s ethnicity was found in the official school records and confirmed during the parent-teacher conferences.

### Participants

Five hundred Roma and non-Roma children from two school districts located in the Transylvania region of Romania participated in our study. The selection of the participants was random, and their participation in this study was voluntary and contingent on the written consent of their parents. The children were all registered in the First grade in one of the 21 participating schools. The schools were selected because their demographic information indicated that many of them had a high percentage of Roma children enrolled. All schools were state funded (like the majority of schools in Romania) and enrollment was not based on the students’ financial status. The Roma and non-Roma children participating in this study were recruited from the same communities, although some schools (and communities) had a higher percentage of Roma children than others.

Out of the 500 participants, 322 were Roma (172 boys, Mage = 89.57 months, *SD* = 5.05) and 178 were non-Roma (89 boys, Mage = 88.22 months, *SD* = 4.18).

### Measures

#### General Intelligence

The General Intelligence score was assessed using Raven’s Colored Progressive Matrices (RCPM) (Raven et al., 1991). The scale includes 36 items with increased complexity, and the responses were coded from 0 to 36. The internal consistency of our data indicated that the measure had a high reliability (Cronbach’s alpha = 0.86).

#### Socio-Economic Status

The socio-economic status data was collected from parents through a questionnaire. The data collected included information about the mother’s education, the father’s education, family income, and living conditions. Each parent’s level of education was coded on a scale from 1 (elementary education) to 9 (doctorate). The two educational indicators correlated strongly (*r* = 0.755, *p* < 0.001) and consequently, we have created a composite score of the parents’ education by calculating the average of the two scores. The income data collected was ranked on a scale from 1 (less than 50 USD/month) to 13 (more than 1000 USD/month). For the living conditions, we have calculated a score reflecting the ratio between the number of family members relative to the number of rooms per household. The scores ranged in our study from 0.5 to 10.

## Results

To answer the first question, we conducted a one-way ANCOVA to test for differences in IQ between Roma and non-Roma students, with age as covariate (see also descriptive statistics in Table 1). There was a significant effect of ethnicity on IQ, after controlling for age *F*(2,473) = 101.05, *p* < 0.001, partial η*^2^* = 0.18. Roma children had lower IQ scores (*M* = 14.87, *SD* = 5.47) than non-Roma children (*M* = 20.44, *SD* = 6.10).

**TABLE 1:** Descriptive statistics.

Variable	Roma children				Non-Roma children			
	*M*	*SD*	Minimum	Maximum	*M*	*SD*	Minimum	Maximum
IQ	14.85	5.45	1.00	33.00	20.40	6.08	4.00	33.00
Parental education	1.91	0.92	1.00	7.00	3.52	1.35	1.00	7.50
Income	4.37	2.65	1.00	12.00	6.99	3.34	1.00	13.00
Living conditions	3.41	1.88	0.50	9.00	2.07	1.26	0.50	10.00

To answer the second question, we first compared the SES indicators of Roma and non-Roma children. The results indicated that the Roma children had parents with a lower level of education *t*(262.68) = −13.81, *p* < 0.001, *d* = 1.47, they lived in households with lower incomes *t*(282.67) = −8.68, *p* < 0.001, *d* = 0.90, and their living conditions were poorer *t*(479.69) = 9.46, *p* < 0.001, *d* = 0.80 as compared to those of their non-Roma peers. Next, we ran a Pearson product-moment correlation to assess the relationship between each SES indicator and the IQ scores of our participants. Results show that there was a moderate positive correlation between IQ and the parents’ education, *r* = 0.494, p < 0.01, and income, *r* = 0.345, *p* < 0.01, as well as a moderate negative correlation between IQ and living conditions, *r* = −0.329, *p* < 0.01. The results supported the hypotheses that (a) the SES of Roma children was lower than that of their non-Roma peers, and (b) the SES can explain important variance in IQ scores. Consequently, we conducted a one-way ANCOVA to test for differences in IQ between Roma and non-Roma students, while controlling for age, parents’ education, living conditions and income as covariates. While the effect size diminished considerably, we still found a significant effect of ethnicity on IQ, *F*(5,431) = 14.78, *p* < 0.001, partial η*^2^* = 0.03. Roma children had lower IQ scores (*M* = 14.87, *SD* = 5.47) than non-Roma children (*M* = 20.44, *SD* = 6.10). Both the parents’ education *F*(5,431) = 32.03, *p* < 0.001, and living conditions *F*(5,431) = 5.53, *p* = 0.02 were found to have an effect on IQ.

The results suggested that some SES indicators (i.e., parents’ education and living conditions) partially mediate the relationship between ethnicity and IQ, but the magnitude of their predictive effect can vary. Therefore, we subsequently conducted a mediation analysis using Process Macro (Hayes, 2017). The socioeconomic indicators were entered as parallel mediators. Results indicated significant indirect effects of ethnicity on IQ through education (*B* = 2.27, *SE* = 0.49, 95% CI = 1.34, 3.28) and living conditions (*B* = 0.46, *SE* = 0.22, 95% CI = 0.02,0.89). Income was not a statistically significant mediator (95% CI = −0.26,0.95). Furthermore, pairwise contrasts suggested a larger indirect effect of ethnicity on IQ through education as compared to the indirect effect through living conditions (*B* = 1.81, *SE* = 0.60, 95% CI = 0.67, 3.02) (see Table 2).

**TABLE 2:** Bootstrapped estimates and confidence intervals for total effects, specific indirect effects, and pairwise contrasts of indirect effect.

	*B*	*SE*	95% CI	
			Lower	Upper
Total direct effect	2.77	0.69	1.41	4.12
**Indirect effects**				
Education	2.27	0.49	1.34	3.28
Living conditions	0.46	0.22	0.02	0.89
Income	0.31	0.30	–0.26	0.95
Total	3.04	0.46	2.18	3.96
**Contrasts**				
Education vs. Living conditions	1.81	0.60	0.67	3.02

## Discussion

The present study aimed to test whether and to what extent Roma children perform more poorly than their non-Roma peers from the same communities on non-verbal tests such as RPM, as well as to measure to what extent socio-economic factors explain test performance.

As anticipated, and in line with previous findings, Roma children performed significantly more poorly than their non-Roma peers on non-verbal abilities tests, and the effect size between the means of the two groups was medium, with ethnicity explaining 18% of the variance of the IQ test performance. Our findings are in line with those of Rushton et al. (2007) and other studies (see above) and suggest that, on average, the Roma perform significantly more poorly than their non-Roma peers on RPM.

The most important facet of our study was accounting for the potential effects of SES indicators on the IQ test performance of Roma children. As anticipated (e.g., Brüggemann, 2012; Dolean et al., 2016, 2019), the SES of Roma children was significantly lower than the SES of their non-Roma peers, with effect sizes ranging from 0.80 to 1.47 SD. These contrasts supported the assumption that the non-verbal abilities of Roma children might be at least partially explained by their low SES. Our further analysis confirmed this hypothesis. The results indicating a strong predictive effect of parent’s education and living conditions on IQ test scores of Roma children are consistent with previous research (Solari and Mare, 2012; Ferguson et al., 2013; Ritchie and Tucker-Drob, 2018). They posit that even the non-verbal IQ tests can be culturally loaded (Kan et al., 2013), and the performance on RPM is dependent on contextual factors (Owen, 1992). They also indicate that Roma children do not have an equal opportunity to perform well on non-verbal IQ tests when compared with their non-Roma peers because their socio-economic circumstances related to parents’ education and living conditions make them more likely to perform poorly on these tests. It was particularly interesting to find that income was not an important predictor of IQ test scores after we accounted for the parents’ education and living conditions. This is surprising, as existing literature claims that income can be strongly associated with cognitive abilities in general (Noble et al., 2015) and IQ scores measured with RPM, in particular (Mani et al., 2013). However, education and income are usually measured under the same construct (SES) and few studies disentangle the two variables as ours does.

When we compared the two ethnic groups after accounting for the SES indicators, we still found a significant effect for ethnicity, although this effect size was small and substantially diminished when compared with our previous analysis. Our results suggest that indeed, some of our measures of socio-economic status captured most of the variance between the two ethnic groups; however, the influence of the variables was not strong enough to completely mediate the effects of ethnicity on the IQ scores. It is very possible that other factors might play an important role in the performance of Roma children on IQ tests, underscoring both the limitations of this study and providing directions for further research.

One of these factors is kindergarten attendance. Several reports indicate that throughout Europe, Roma children attend kindergarten less frequently than their non-Roma peers (European Union Agency for Fundamental Rights [FRA], 2011). For instance, the report indicated that in Romania (where kindergarten attendance was not mandatory at the time the data was collected), the percentage of non-Roma children attending kindergarten was twice that of their Roma peers. Given that some skills measured by RPM can be dependent on abilities that are formed in kindergarten such as print concept, stamina in test-taking situations, self-regulation abilities, following directions and manipulating abstract geometrical shapes, it is fair to assume that the kindergarten attendance (or lack thereof) of the children in our study could have had a significant effect on their test performance. Furthermore, the potential differences between Roma and non-Roma kindergarten attendance rates could explain a general familiarity with test-taking conditions, in that children who attended kindergarten may have been more likely to perform better on any test, including RPM (Hausknecht et al., 2007). For instance, two recent longitudinal studies (Dolean et al., 2019; Lervåg et al., 2019) indicated that the school attendance rate of Roma children explained an important variance in performance on vocabulary and reading tests beyond cognitive and non-cognitive factors, and partially mediated the effects of SES on children’ test performance. Thus, not accounting for kindergarten attendance is a limitation of this study, and further research is needed to explore the extent to which the kindergarten attendance rate of Roma children could explain their performance on tests administered at the beginning of their schooling experience.

Other variables that might explain the differences in performance of the two groups on RPM that are not accounted for in this study are related to cultural aspects that could influence Roma children’s attitudes toward school and their motivation in test-taking situations. Several studies underscore important cultural differences between Roma and their non-Roma peers from the same communities, in that, on average, Roma do not seem to place as great a value on education (and the opportunities offered by schools) as their non-Roma peers (Cretan and Turnock, 2008; Kosko, 2012). This phenomenon can be explained by the fact that Roma typically have more difficulties than non-Roma in translating the advantages of schooling into gainful employment and realizing the positive outcome of such a laborious effort. These cultural differences could explain an important variance in test-taking situations, as research has shown that an important variance in the performance of students on cognitive tests is explained by their motivation and attitude toward tests (Penk and Richter, 2017; Gignac et al., 2019). Thus, not accounting for the potential differences in motivation and attitude toward school between Roma and non-Roma children is another limitation of this study.

Finally, the samples of Roma and non-Roma children were not balanced. Although unequally sized groups are common in research, there is a risk that the underrepresented group (non-Roma) might lose statistical power. Given this limitation, the interpretation of our results needs to be treated with caution.

## Implications

For many years Roma minority children have been denied access to high-quality education based on their underperformance on tests that measure their general non-verbal abilities, such as RPM. Such poor performance could have potentially fueled a downward social comparison (Festinger, 1954) that made educational stakeholders feel justified in the exclusionary practices of Roma children simply because they do not meet the standards of the non-Roma majority. The results of this study show that such tests do not measure the true, unbiased general cognitive performance of Roma children and point out that factors associated with the parents’ education and living conditions (but not income) explain much of their underperformance on this test. The results confirm most of the assumptions made by Rushton et al. (2007). They also indicate that non-verbal IQ tests such as RPM can “underestimate the true Roma population mean” (Rushton et al., 2007, p. 10), and therefore, cannot be fairly used to assess the potential of Roma children without accounting for their socio-economic background. Consequently, in order to address the poor performance of Roma children on IQ tests, a more comprehensive assessment battery needs to be used that would account for the socio-economic factors affecting the development of Roma children. Furthermore, given that IQ is a strong predictor of educational achievement (Ritchie and Tucker-Drob, 2018), our data indicate that a strong focus of educational policies geared to help Roma children perform well in school should be placed on the enhancement of the lifelong learning experiences of Roma parents, as well as the improvement of the living conditions of Roma children.

## Data Availability Statement

The datasets generated for this study are available on request to the corresponding author.

## Ethics Statement

The manuscript adheres to ethical guidelines in the APA Code of Conduct and Ethics Guidelines from Romania. The studies involving human participants were reviewed and approved by Babes-Bolyai University Research Ethic Committee. Written informed consent to participate in this study was provided by the participants’ legal guardian/next of kin.

## Author Contributions

DD contributed with the data collection and manuscript framework and writing. AC contributed with the statistical analysis and reporting of the results. All authors contributed to the article and approved the submitted version.

## Conflict of Interest

The authors declare that the research was conducted in the absence of any commercial or financial relationships that could be construed as a potential conflict of interest.
